# Exploration of the causal associations between circulating inflammatory proteins, immune cells, and neuromyelitis optica spectrum disorder: a bidirectional Mendelian randomization study and mediation analysis

**DOI:** 10.3389/fnagi.2024.1394738

**Published:** 2024-04-26

**Authors:** Zhiqing Chen, Yujin Guo, Huaiyu Sun, Wuqiong Zhang, Shuai Hou, Yu Guo, Xiaohui Ma, Hongmei Meng

**Affiliations:** ^1^Department of Neurology and Neuroscience Center, The First Hospital of Jilin University, Changchun, China; ^2^Department of Ophthalmology, The Second Hospital of Jilin University, Changchun, China

**Keywords:** inflammatory proteins, immune cells, neuroinflammation, neuromyelitis optica spectrum disorder, Mendelian randomization, mediation analysis

## Abstract

**Background:**

An increasing body of research has demonstrated a robust correlation between circulating inflammatory proteins and neuromyelitis optica spectrum disorders (NMOSD). However, whether this association is causal or whether immune cells act as mediators currently remains unclear.

**Methods:**

We employed bidirectional two-sample Mendelian randomization (TSMR) analysis to examine the potential causal association between circulating inflammatory proteins, immune cells, and NMOSD using data from genome-wide association studies (GWAS). Five different methods for Mendelian randomization analyses were applied, with the inverse variance-weighted (IVW) method being the primary approach. Sensitivity analyses were further performed to assess the presence of horizontal pleiotropy and heterogeneity in the results. Finally, a two-step Mendelian randomization (MR) design was employed to examine the potential mediating effects of immune cells.

**Results:**

A notable causal relationship was observed between three circulating inflammatory proteins (CSF-1, IL-24, and TNFRSF9) and genetically predicted NMOSD. Furthermore, two immune cell phenotypes, genetically predicted CD8 on naive CD8+ T cells, and Hematopoietic Stem Cell Absolute Count were negatively and positively associated with genetically predicted NMOSD, respectively, although they did not appear to function as mediators.

**Conclusion:**

Circulating inflammatory proteins and immune cells are causally associated with NMOSD. Immune cells do not appear to mediate the pathway linking circulating inflammatory proteins to NMOSD.

## 1 Introduction

Neuromyelitis optica spectrum disorder (NMOSD) is an autoimmune-induced inflammatory demyelinating (IDD) disease of the central nervous system (CNS) characterized by astrocyte dysfunction and loss (Shi et al., 2022; Qin et al., 2024). NMOSD primarily affects the optic nerves and spinal cord (Carnero Contentti and Correale, 2021), with recurrent episodes potentially resulting in severe consequences including permanent blindness, paralysis, and mortality (Weinshenker et al., 2006; Mealy et al., 2012; Shi et al., 2022).

Aquaporin-4 (AQP4), a key target antigen for autoimmunity in NMOSD, is localized to water channels (Carnero Contentti and Correale, 2021). AQP4 antibodies (AQP4-abs) have been identified as key differentiators between NMOSD and other CNS demyelinating conditions, such as multiple sclerosis (Wingerchuk et al., 2007; Jasiak-Zatonska et al., 2016). However, the cause of this disease in AQP4 antibody-negative patients remains unclear (Yamamura et al., 2019). Steroid hormones, immunosuppressive agents, and monoclonal antibodies are commonly used in the clinical treatment of NMOSD. However, these drugs only partially suppress acute-phase inflammatory episodes, and do not completely inhibit relapse or CNS damage (Carnero Contentti et al., 2020). However, effective methods for improving regeneration and functional recovery are still lacking (Carnero Contentti and Correale, 2021), and further research is required to improve our understanding of these underlying mechanisms.

In NMOSD, immune components interact with peripheral tissues and the CNS. Most AQP4- abs are produced by peripheral lymphocytes (Carnero Contentti and Correale, 2021). In the context of systemic inflammation, peripherally-produced AQP4-abs can damage the CNS (Bennett et al., 2009; Carnero Contentti and Correale, 2021). Previous studies have reported that certain peripheral inflammatory cytokines, such as IL-6 and other Th17-associated cytokines, can disrupt the blood–brain barrier (BBB). This facilitate the transfer of AQP4-abs and inflammatory mediators to the CNS (Liu et al., 2022; Chu et al., 2023). AQP4-abs that enter the CNS bind to AQP4 located at the terminus of astrocytes. This binding promotes neuroinflammation by activating the complement system and microglia, thereby facilitating the disruption of astrocyte membranes (Hinson et al., 2007; Fujihara et al., 2020). Ultimately, this leads to myelin damage and neuronal loss (Kawachi and Lassmann, 2017; Carnero Contentti and Correale, 2021). Moreover, the presence of an inflammatory microenvironment within the CNS leads to increased cerebrovascular permeability and BBB disruption, facilitating the infiltration of effector immune cells and their associated inflammatory mediators into the CNS (Vincent et al., 2008; Carnero Contentti and Correale, 2021). This feedback loop can exacerbate neuroinflammation, leading to further demyelination.

The critical role of inflammatory cytokines in the pathophysiology of NMOSD is well established (Fujihara et al., 2020). However, the interplay of immune components between CNS and peripheral tissues owing to BBB disruption is complex. Further, whether systemic inflammation is the cause or a downstream effect of NMOSD remains unclear. The pathogenesis of NMOSD involves the generation of harmful antibodies by B cells, in addition to damage to the myelin through the infiltration of neutrophils and lymphocytes. Meanwhile, pro-inflammatory cytokines play crucial roles in this immune response, including supporting the survival of plasma cells (Fujihara et al., 2020), inducing the production of AQP4-abs (Kowarik et al., 2015), impairing the integrity and function of the BBB (Takeshita et al., 2017), and promoting the polarization of Th17 cells (Dos Passos et al., 2016). As such, circulating immune cells may act as intermediaries in the causal association between systemic inflammatory proteins and NMOSD.

Mendelian randomization (MR) is an analytical technique that employs genetic variation as instrumental variables (IVs) to investigate causal links between variables and illnesses (Wootton et al., 2020; Wang et al., 2023). Unlike traditional observational studies, MR is less affected by confounding factors and reverse causation (Burgess et al., 2015), as genetic variations occur during conception before disease onset, following a random assignment pattern during meiosis (Swerdlow et al., 2016). In this study, we conducted a bidirectional MR analysis with two samples and a mediation analysis using data from recent genome-wide association studies (GWAS) to evaluate the causal relationship and direction of the association between systemic inflammatory proteins and NMOSD. Furthermore, we investigated the potential role of immune cells as mediators in the pathway from inflammatory proteins to NMOSD.

## 2 Materials and methods

### 2.1 Study design

**Figure 1:**
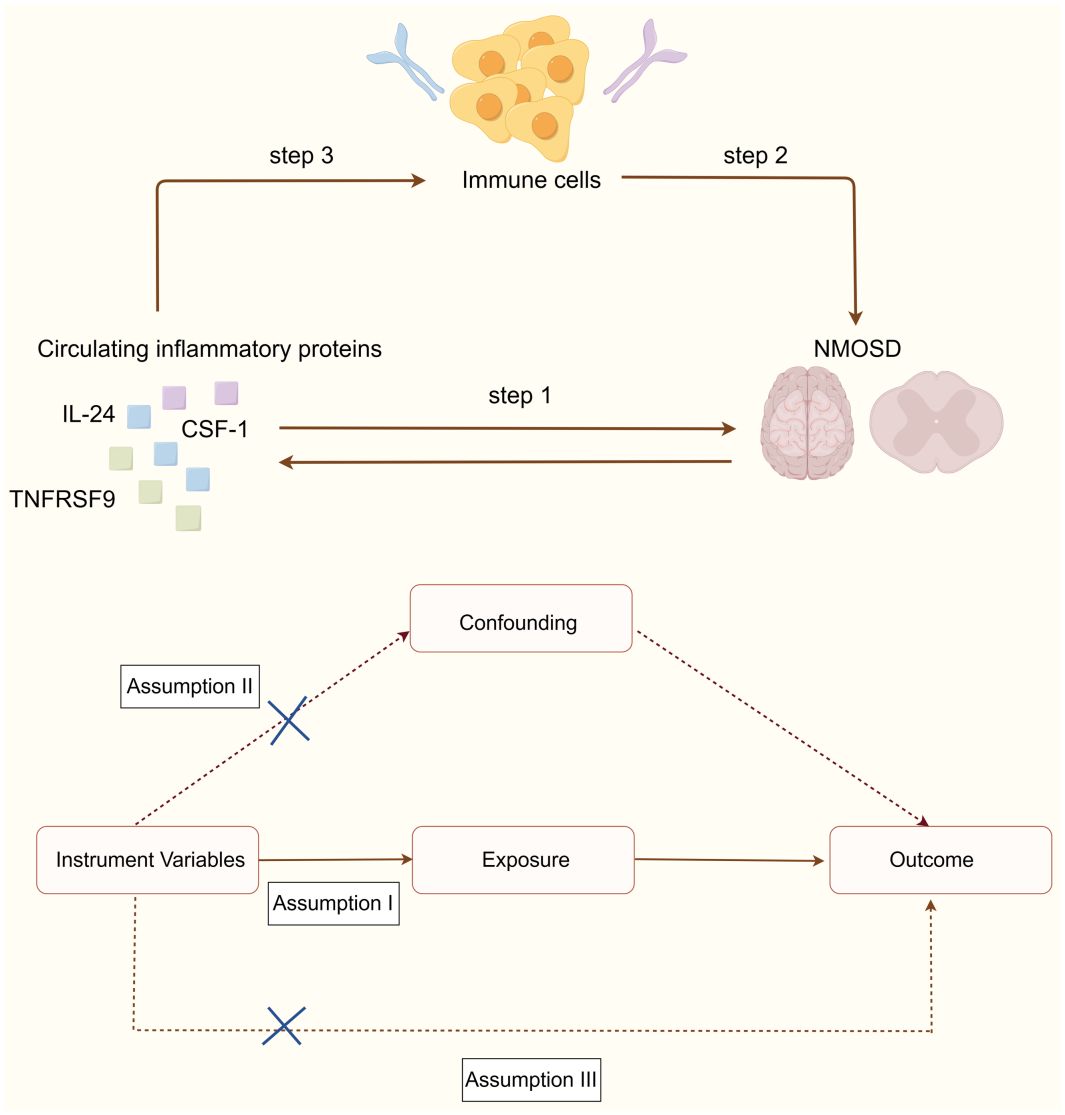
Schematic diagrams depicting the experimental design.

MR study was used to assess the causal association between 91 circulating inflammatory proteins and NMOSD. The analysis was performed in three sequential stages, as shown in Figure 1. First, the causal effects of the 91 inflammatory proteins on NMOSD were examined (step 1). Subsequently, the causal effects of 731 immune cells on NMOSD were examined (step 2). Finally, the immune cell-mediated effects of circulating inflammatory proteins on the pathogenic pathway of NMOSD were examined (step 3). In this research, the causal relationship was evaluated using single-nucleotide polymorphisms (SNPs) as IVs. The MR study design needed to satisfy three fundamental assumptions: (1) a strong association exists between the IVs and exposure, (2) IVs are independent of any confounding factors, and (3) IVs can influence the outcome solely through exposure.

The studies included in our analysis were authorized by the appropriate institutional review boards, and participants provided informed consent to participate. Ethical approval was not required for this study as all data were publicly available.

### 2.2 Data source

Genetic data on circulating inflammatory proteins were obtained from the latest GWAS meta-analysis, which included 11 study cohorts comprising 14,824 participants of European ancestry. This study analyzed the genome-wide genetic data of 91 inflammatory proteins using the Olink-Targeted Inflammation Panel. The GWAS summary statistics for each inflammatory protein are publicly available in the GWAS catalogue (accession numbers GCST90274758: GCST90274848) (Zhao et al., 2023). Genetic data for immune cells were obtained from a previous GWAS (GCST0001391-GCST0002121), that investigated 731 immune cell traits (Orrù et al., 2020).

The GWAS dataset for genetic prediction of NMOSD risk was used as the outcome data. The study was performed in accordance with the 2015 NMOSD diagnostic criteria and included 1,459 subjects, including: 132 AQP4-IgG seropositive NMOSD, 83 AQP4-IgG seronegative NMOSD, and 1,244 control subjects (Wingerchuk et al., 2015; Estrada et al., 2018).

### 2.3 IVs selection

As only a limited number of SNPs met the criteria for genome-wide significance, we established a cutoff value of 5 × 10^−6^ to identify IVs related to inflammatory proteins. This method to appropriately relax IV selection thresholds has been widely used in previous studies (Cao et al., 2022; Wang et al., 2023). Subsequently, genetic differences were grouped together in a 10,000-kb range based on linkage disequilibrium (LD), with a cut-off of *r*^2^ = 0.001 for grouping. Palindromic SNPs and SNPs strongly associated with outcome-associated SNPs were excluded from the study, and the F-statistic for each SNP was computed to determine its statistical strength in relation to exposure. If the genetic instrument is weak, it may not fully explain the association between phenotypic variations and outcomes, which could introduce bias (Bound et al., 1995). However, the extent of bias resulting from a weak instrument depends on the strength of the F-statistic of the IV. When F equals 10, the mean estimate of the IV estimator has a relative bias of 10% compared with the true value (Lawlor et al., 2008; Burgess et al., 2011). To reduce the bias resulting from weak IVs, only SNPs with *F* > 10 were retained for subsequent MR analyses (Ji et al., 2024). Furthermore, SNPs linked to immune cells were chosen at a significance threshold of *p* < 1 × 10^−5^. Supplementary Tables S1–S3 presents comprehensive details of the significant IVs that were ultimately selected.

### 2.4 Statistical analyses

Five MR methods, inverse variance-weighted (IVW), MR-Egger, simple mode, weighted median, and weighted mode, were employed to evaluate the potential causal link between the levels of inflammatory proteins in the circulation and the risk of NMOSD. The primary IVW method for MR analysis is effective at estimating causal effects and addressing potential heterogeneity (Burgess et al., 2015). The Cochran’s Q test was used to measure the heterogeneity of each SNP. When there was heterogeneity in the Wald ratio estimates of the SNPs (*p* < 0.05), a random-effects IVW model was used; otherwise, a fixed-effects IVW model was used (Zhang et al., 2023). Various techniques have been employed to identify potential horizontal pleiotropy, including the MR-PRESSO global test and MR-Egger intercept test, as these the primary approaches used to detect the existence of horizontal pleiotropy in IVs. In cases where both methods have *p*-values greater than 0.05, we can conclude that horizontal pleiotropy is absent (Burgess and Thompson, 2017). The leave-one-out method was used to assess the effects of individual SNP drivers on the random estimates. Scatter and funnel plots were plotted to further verify the robustness of the results. Further, the Steiger directionality test and reverse MR analysis were used to determine the direction of causality between exposure and outcome (Zhong et al., 2024). Reverse causality was assumed when an anomaly was detected. To reduce the likelihood of type I errors during multiple hypothesis testing, we applied a false discovery rate (FDR) technique to adjust the IVW results after per-analysis of the 91 inflammatory proteins with a single outcome. A pFDR value of less than 0.05 indicates a significant causal relationship (Zhang et al., 2023). To identify potential immune cell characteristics that act as intermediaries between inflammatory proteins and NMOSD, we conducted a two-step MR analysis of notable inflammatory proteins to separate the direct and indirect effects of immune cells and inflammatory proteins. The first step investigated the causal effect of immune cells on NMOSD, and included significant immune cell phenotypes as potential mediators. In the second phase, the impact of inflammatory proteins on intermediate mediators was evaluated.

Data were analyzed using the TwoSampleMR (version 0.5.8) and MR-PRESSO (version 1.0) packages in R (version 4.3.2).

## 3 Results

### 3.1 Effect of the 91 inflammatory proteins on NMOSD

**Figure 2:**
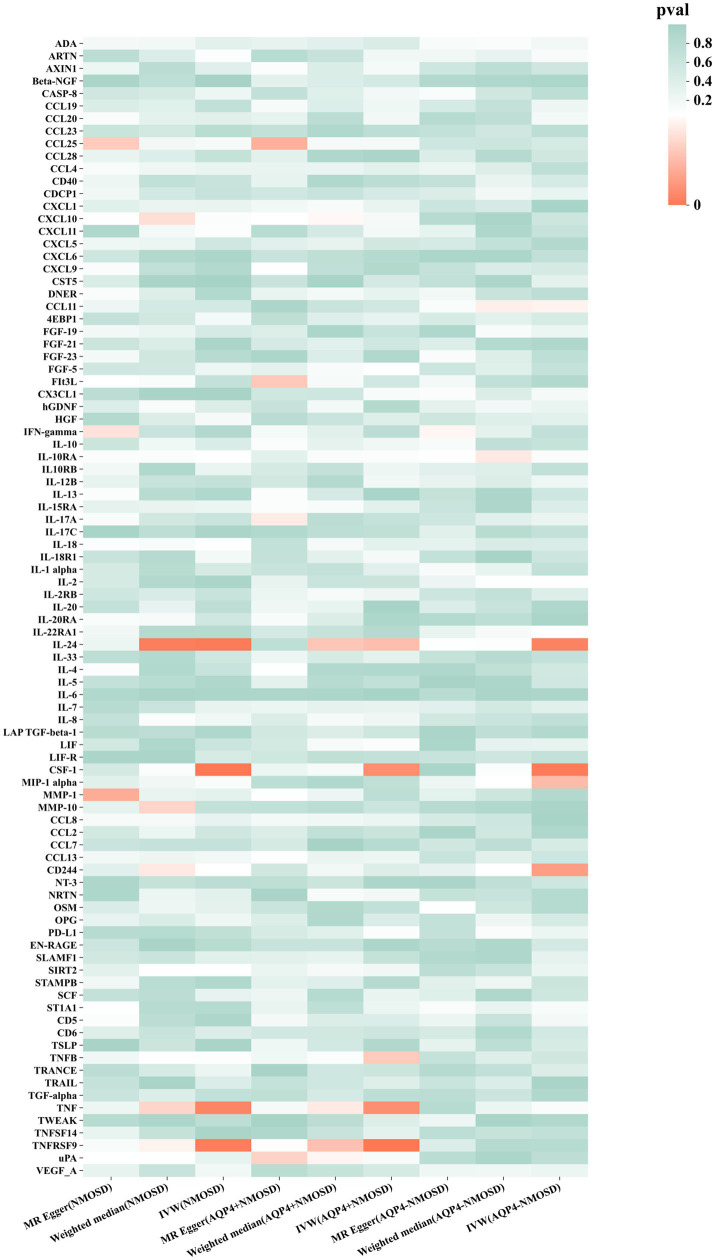
Mendelian randomization of associations between circulating inflammatory proteins and NMOSD.

**Figure 3:**
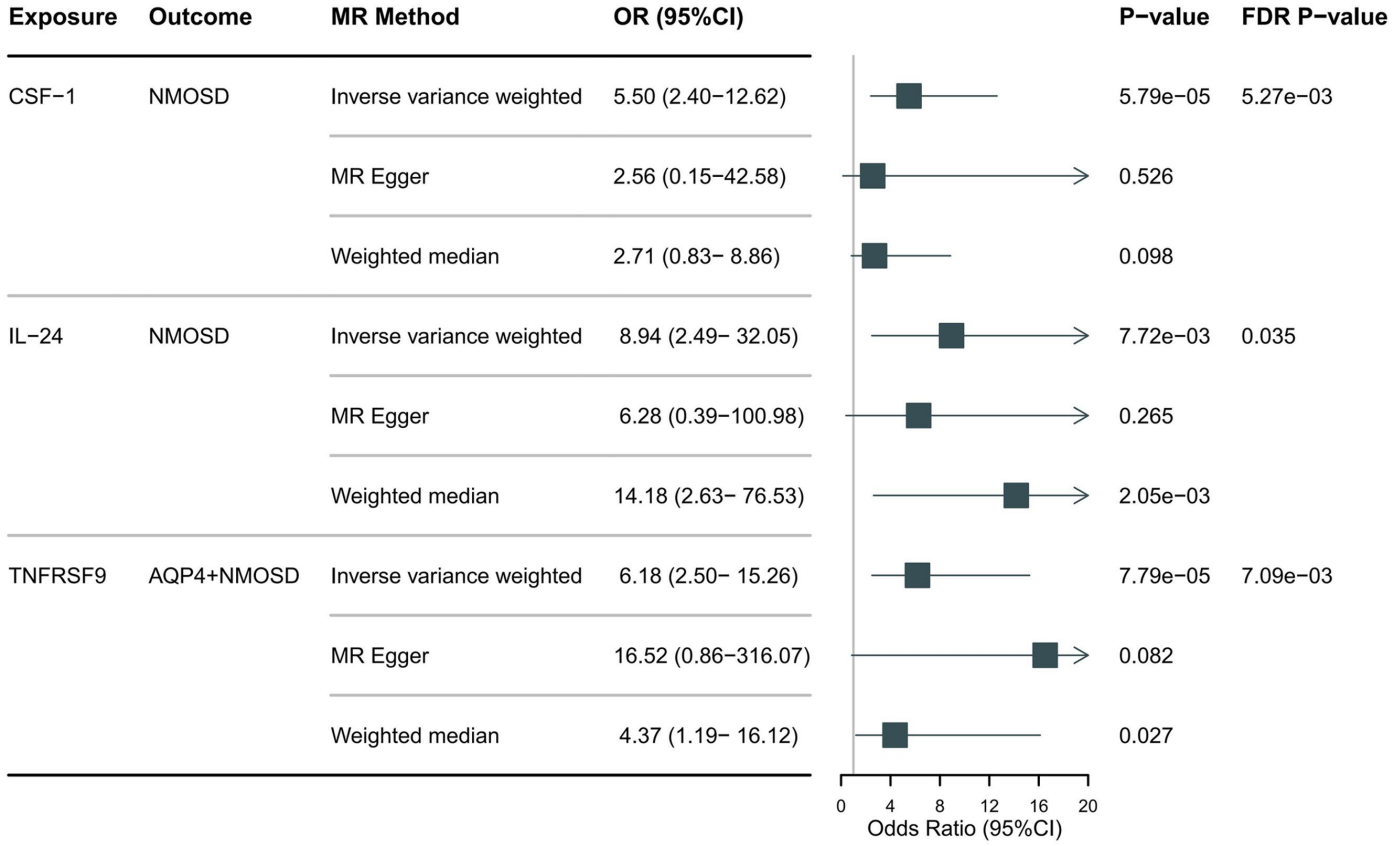
Forest plot showing the causal relationship between circulating inflammatory proteins and NMOSD (pFDR <0.05).

Preliminary IVW identification (Figure 2 and Supplementary Table S4) identified 14 trait pairs with nominal causal associations (*p* < 0.05), of which four, five, and five inflammatory proteins associated with the risk of all NMOSD, AQP4 + NMOSD, and AQP4-NMOSD, respectively, were identified. After FDR correction, we observed that the three inflammatory proteins had significant pathogenic associations with NMOSD and its subtypes (FDR < 0.05). The result of the MR analysis showed a significant positive correlation between the genetically predicted macrophage colony-stimulating factor 1 (CSF-1) (OR: 5.50; 95%CI: 2.40–12.62; *p* = 5.79 × 10^−5^; pFDR = 0.005; IVW) and IL-24 (OR: 8.94; 95%CI: 2.49–32.05; *p* = 0.007; pFDR = 0.035; IVW) with NMOSD (Figure 3). Tumor necrosis factor receptor superfamily member 9 (TNFRSF9) (OR: 6.18; 95%CI: 2.50–15.26; *p* = 7.79 × 10^−5^; pFDR = 0.007; IVW) also showed a remarkable positive correlation with AQP4 + NMOSD (Figure 3). Furthermore, the direction of the β estimates, which reflects the causal relationship between these three sets of traits, was consistent across the five MR methods, adding to the robustness of our findings. The results of more detailed MR analyses are presented in Supplementary Table S5. The results of the sensitivity analysis are presented in Supplementary Table S6. We focused on identifying heterogeneity and horizontal pleiotropy among the inflammatory proteins that showed significant causal associations with the outcome. This analysis revealed no heterogeneity among the data, as the Cochran’s test was not statistically significant (*p* > 0.05). Both the MR-PRESSO global test and the MR-Egger intercept test yielded *p*-values above the 0.05 threshold, indicating no significant horizontal pleiotropy bias. Leave-one-out plots showed that the MR results were not affected by any single SNP (Supplementary Figures S5, S6). Scatter and funnel plots confirmed the stability of the results (Supplementary Figures S1–S4). These results consistently supported a significant causal relationship between the genetic prediction results.

### 3.2 Reverse analysis and Steiger directionality test

We further performed reverse MR analysis and Steiger directionality tests on NMOSD and its subtypes. No significant reverse causality was found, suggesting that NMOSD did not have a causal effect on circulating inflammatory proteins. The results of the reverse analyses and Steiger directionality tests are shown in Supplementary Tables S7–S9.

### 3.3 Mediation analysis

**Figure 4:**
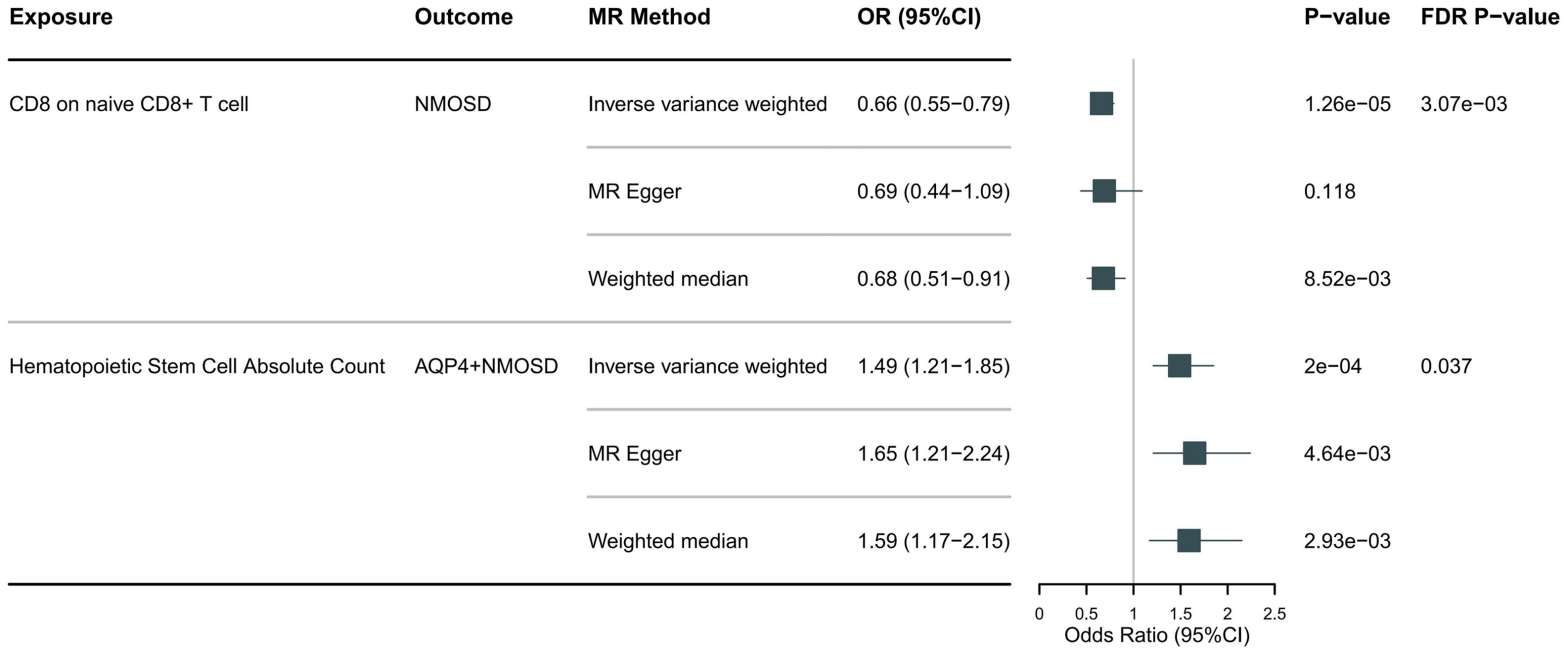
Forest plot showing the causal relationship between circulating immune cells and NMOSD (pFDR <0.05).

**Figure 5:**
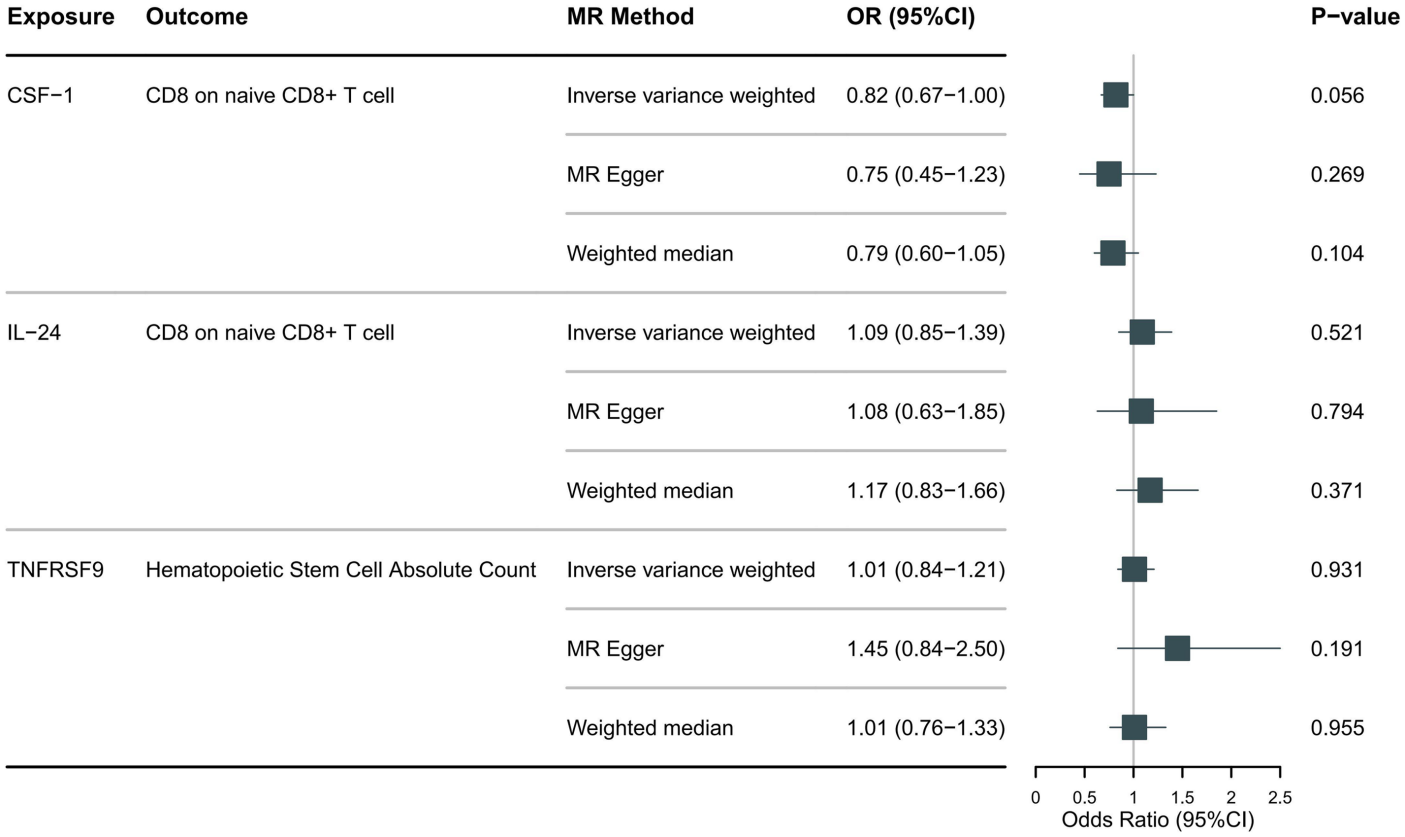
Forest plot showing the causal relationship between identified circulating inflammatory proteins and immune cells.

Following the foundational principles of the two-step MR approach for mediation analysis, we selected immune cell phenotypes that demonstrated statistically significant causal relationships with the outcome (FDR < 0.05) as representative immune cells for subsequent mediation analysis. After adjusting for the FDR, the two immune cell phenotypes were found to exhibit significant causal associations with NMOSD. As shown in Figure 4, genetically predicted CD8 on naive CD8+ T cells (OR: 0.66; 95% CI: 0.55–0.79; *p* = 1.26 × 10^−5^; pFDR = 0.003; IVW) was found to reduce the risk of NMOSD. Meanwhile, genetically predicted Hematopoietic Stem Cell Absolute Count (OR: 1.49; 95% CI: 1.21–1.85; *p* = 2 × 10^−4^; pFDR = 0.037; IVW) was associated with an increased risk of AQP4 + NMOSD. In the present study, both circulating inflammatory proteins and immune cells were found to be involved in NMOSD. Immune cells appear to mediate the pathway between inflammatory proteins and NMOSD. One requirement for a mediating role is that inflammatory proteins are significantly associated with immune cells. However, our results showed no causal relationship between circulating inflammatory proteins associated with NMOSD and immune cells associated with NMOSD (Figure 5), suggesting that immune cells do not act as mediators in pathways involving circulating inflammatory proteins and NMOSD.

## 4 Discussion

To our knowledge, this is the first study to comprehensively assess the cause-and-effect relationship between 91 circulating inflammatory proteins and NMOSD using a bidirectional two-sample Mendelian randomization (TSMR) design. This study revealed that genetically forecasted CSF-1, IL-24, and TNFRSF9 levels were notably linked to an increased risk of NMOSD development. However, there was no evidence to suggest that NMOSD affects the levels of inflammatory proteins in circulation. To explore the potential underlying mechanisms, we performed mediation analyses using TSMR, and identified two immune cell phenotypes that were significantly associated with NMOSD, but did not act as intermediate mediators of circulating inflammatory proteins associated with NMOSD pathogenesis.

Colony Stimulating Factor 1 receptor (CSF-1R) is a cell-surface receptor tyrosine kinase that binds primarily to CSF-1 (Percin et al., 2018). In the context of inflammation, CSF-1R may indirectly contribute to pro-inflammatory responses by promoting the survival and proliferation of inflammatory myeloid cells (Hagan et al., 2020). Additionally, CSF-1R is expressed in myeloid cells within the CNS, including microglia and monocytes (Hume and MacDonald, 2012). Multiple studies have verified the role of the CSF-1/CSF-1R axis in the development of inflammatory and demyelinating disorders that affect the CNS (Hwang et al., 2022; Xiang et al., 2023). Groh et al. observed myelin damage in a CSF-1-mediated model of Charcot–Marie–Tooth disease type 1X (CMT1X) as a result of continuous activation of microglia and macrophages (Groh et al., 2012). In an animal model of multiple sclerosis, treatment with anti-CSF-1 was shown to selectively deplete myeloid cells in the inflammatory regions of the CNS. This resulted in a reduction in inflammation, the size of demyelinating lesions in white matter regions, and microglial activation in the grey matter (Hwang et al., 2022). Although there is a paucity of direct research on CSF-1 in individuals with NMOSD, a multitude of similar immunological pathways exist in demyelinating disorders. In NMOSD, microglia-derived C1q has been shown to have a dual effect of directly damaging oligodendrocytes and neurons (Chen et al., 2020) and activating the complement cascade response (Moinfar and Zamvil, 2020), leading to a cascade of subsequent events. It has further been hypothesized that the upregulation of CSF-1 expression to facilitate the expansion of microglia at the site of inflammation may play a crucial role in the immunopathogenesis of NMOSD. Considering the significance of CSF-1 in CNS demyelinating diseases, further validation using comprehensive clinical data is warranted to elucidate the relationship between CSF-1 levels and NMOSD.

IL-24 is a proinflammatory cytokine associated with tissue inflammation and autoimmune diseases (Persaud et al., 2016). It is also expressed in the CNS and is involved in neuroinflammation (Dayton et al., 2021; Zhang et al., 2022). Biologically, IL-24 belongs to the interleukin-20 receptor (IL-20R) cytokine family and binds to IL-20RB, forming dimers with IL-20RA or IL-22RA1 (Chen J. et al., 2018). Further research has shown that it induces immune cells to express chemokines and VEGFA (Chen G. et al., 2018; Dayton et al., 2021), subsequently resulting in BBB breakdown and immune component leakage (Dayton et al., 2021). Our statistical analysis revealed a significant association between IL-24 levels and the risk of developing NMOSD. There is no existing literature on this relationship. However, one study utilizing an experimental autoimmune encephalomyelitis (EAE) model, a model for multiple sclerosis, reached conclusions similar to those of our findings. In the EAE model, mice with ablation of the IL-20RB subunit (IL-20RB−/−) showed improvements in disease severity, time to first attack, and neuropathological changes compared to wild-type mice. Spinal cord IL-24 levels were also found to be significantly higher in wild-type mice than in IL-20RB−/− mice, suggesting that IL-24 plays a role in CNS demyelination diseases (Dayton et al., 2021). In addition, one study showed that IL-24 is involved in the inflammatory mechanism of nerve injury-induced neuropathic pain and that its pathogenic process may be related to CD4 T-cell infiltration (Cai et al., 2024). However, the precise pathogenic mechanism of IL-24 in NMOSD remains unclear. Nevertheless, studies have shown elevated expression of IL-20R in human brain microvascular endothelial cells under inflammatory conditions. It has further been postulated that the interaction between IL-24 and IL-20R may induce the extravasation of immune components by disrupting the parietal-basal polarity within the microvascular BBB (Dayton et al., 2021). This proposed mechanism of BBB disruption could potentially account for the pathogenic effects of IL-24 on NMOSD.

CD137 (TNFRSF9), a member of the tumor necrosis factor receptor family (Wang et al., 2018), serves as a robust growth factor for myeloid cells, and is expressed in both T and vascular endothelial cells. The interaction between CD137 and its ligand CD137L initiates bidirectional signaling (Eissner et al., 2004), leading to the activation of antigen-specific T cells and the polarization of helper T cells (Wong and Schwarz, 2020). Reverse signalling induces the proliferation and differentiation of myeloid cells and the secretion of pro-inflammatory factors, thereby triggering an inflammatory cascade (Langstein et al., 1998; Yeo et al., 2012). Microglia are the most common myeloid cells in the CNS and are activated through reverse signalling pathways. CD137/CD137L signalling activates microglial cells to induce oligodendrocyte cell death through the production of reactive oxygen species, a pathway that has been shown to occur in multiple sclerosis. Gómez et al. found that CD137L knockout mice were significantly more resistant to EAE-induced demyelination (Yeo et al., 2012). A crucial aspect of the pathogenesis of NMOSD is the targeted destruction of oligodendrocytes by activated microglia, which results in subsequent myelin damage. In the context of our findings, the role of TNFRSF9 in the pathogenesis of NMOSD may depend on microglial damage to the axonal myelin.

This study found that CD8 expression on naive CD8+ T cells is associated with a reduced risk of NMOSD. Yang et al. found that the frequency of naïve CD8+ T cells in the peripheral immune cell circulation of patients with NMOSD was significantly reduced, with the number increasing after immunotherapy (Shi et al., 2021). The downregulation of naïve CD8+ T cells in secondary lymphoid tissues such as the bone marrow with organismal aging leads to a decrease in multiple immune reservoirs, which may explain the increased susceptibility to NMOSD in the elderly (Montecino-Rodriguez et al., 2013). Interestingly, senescent cells in a state of cell cycle arrest have a high capacity to secrete proinflammatory factors (Sudo et al., 2000; Coppé et al., 2010), creating an inflammatory environment that negatively affects the migration and functional maturation of naïve lymphocytes in secondary lymphoid tissues (Weng et al., 2009). However, the role of inflammatory proteins in naïve CD8+ T cells was not confirmed in this study. In addition, statistical analyses showed that increased absolute levels of hematopoietic stem cells might increase the risk of AQP4+ NMOSD. A dysfunctional immune system is closely associated with stem cells. Destruction of the host hematopoietic system by chemotherapy and reconstitution of the immune system by hematopoietic stem cell transplantation have emerged as novel therapeutic strategies for refractory NMOSD (Burt et al., 2019; Ceglie et al., 2020).

This MR study provides genetic evidence for a causal relationship between three specific inflammatory proteins and two immune cell phenotypes in NMOSD. These results contribute to our understanding of the relationship between inflammation and CNS demyelinating diseases. Unfortunately, our findings do not support the hypothesis that circulating immune cells mediate the pathogenic pathway of inflammatory proteins in NMOSD. There are three possible reasons. For this First, fluctuations in the peripheral blood levels of inflammatory proteins and immune cells may not consistently reflect alterations in the cerebrospinal fluid. Inflammatory proteins and immune cells on both sides of the BBB may have synergistic or antagonistic effects, which could hinder our ability to explain the correlation between circulating inflammatory proteins and NMOSD by identifying simple mediating substances. Second, the present study only included inflammatory proteins and immune cells that had a significant causal effect on NMOSD in the mediation analysis. The strict FDR correction criteria also limited the number of variables included in the mediation analysis. Finally, the genetically-predicted immune cell phenotype is a quantitative trait. Our research only refutes the notion that changes in the number of immune cell phenotypes play a mediating role. However, disruption of the BBB, activation of the complement cascade, infiltration of multiple immune cells, and interactions among diverse immune components are all also potential mechanisms through which circulating inflammatory proteins may elevate an individual’s susceptibility to NMOSD. Further research into the relationship between systemic inflammation and neuroinflammation in NMOSD episodes, including their dynamics and interactions within the context of the disease, could provide valuable insights into NMOSD.

While MR studies offer advantages in terms of reduced confounding and reverse causality compared to traditional observational studies, there are still limitations to consider. One limitation is the reliance on a limited number of SNPs identified at the genome-wide level, which forced us to use relatively lenient thresholds to select the IVs. Despite efforts to ensure a minimum *F*-statistic of >10 for each SNP, the potential for bias due to weak IVs cannot be excluded. Furthermore, Steiger’s test revealed a valid causal relationship between inflammatory proteins and NMOSD. The Steiger test compares the proportion of variance explained by the exposure SNPs vs. the outcome SNPs, thus supporting the accuracy of our results obtained by relaxing the threshold for selecting IVs (Cai et al., 2022). Second, all the participants in this study were Europeans, which minimized population heterogeneity. However, this limits the generalizability of the findings, even though the underlying biological associations between circulating inflammatory proteins and NMOSD should be similar across different populations. Therefore, future studies are necessary to validate these findings in other populations. Thirdly, the MR analysis may not fully address the issue of horizontal pleiotropy, although multiple sensitivity analyses have been used to mitigate this concern. Finally, previous studies on the inflammatory mechanisms involved in the development of demyelinating diseases have focused on MS. The absence of conclusive literature linking the specific inflammatory proteins identified in our studies to NMOSD necessitates additional validation through randomized controlled trials.

## 5 Conclusion

In summary, this study thoroughly examined the causal links between circulating inflammatory proteins, immune cells, and NMOSD and identified causal links between three genetically predicted inflammatory proteins and two immune cell phenotypes with NMOSD. Although we did not identify immune cells as mediators of the causal relationship between inflammatory proteins and NMOSD, these associations could nevertheless serve as valuable biomarkers and potential targets for understanding the biological mechanisms underlying NMOSD, as well as in the development of new therapies.

## Data availability statement

The original contributions presented in the study are included in the article/Supplementary material, further inquiries can be directed to the corresponding author.

## Author contributions

ZC: Supervision, Writing – original draft, Writing – review & editing. YujG: Data curation, Writing – original draft. HS: Data curation, Writing – original draft. WZ: Methodology, Writing – original draft. SH: Methodology, Writing – original draft. YuG: Methodology, Writing – original draft. XM: Methodology, Writing – original draft. HM: Supervision, Writing – review & editing.
