# Supplementary material for: Exploration of the causal associations between circulating inflammatory proteins, immune cells, and neuromyelitis optica spectrum disorder: a bidirectional Mendelian randomization study and mediation analysis
Source: Front Aging Neurosci. 2024 Apr 26;16:1394738. doi: 10.3389/fnagi.2024.1394738 (PMC11088236; doi:10.3389/fnagi.2024.1394738)
Supplement: Supplementary file 1 [file Data_Sheet_1.docx]

Supplementary Material

### Exploration of the causal associations between circulating inflammatory proteins, immune cells, and neuromyelitis optica spectrum disorder: A bidirectional Mendelian randomization study and mediation analysis

# Supplementary Tables

Supplementary Table 1. The instrumental SNP variables for circulating inflammatory proteins on NMOSD.

Supplementary Table 2. The instrumental SNP variables immune cell phenotypes on NMOSD.

Supplementary Table 3. The instrumental SNP variables for circulating inflammatory proteins on immune cell phenotypes.

Supplementary Table S4: MR primary analysis of 91 inflammatory proteins and NMOSD risk.

Supplementary Table S5: MR analysis of 91 inflammatory proteins and NMOSD risk.

Supplementary Table S6: Sensitivity analysis results of causal effects of inflammatory proteins on NMOSD.

Supplementary Table S7: MR Steiger analysis of the association between 91 inflammatory proteins and NMOSD risk.

Supplementary Table S8:MR analysis results of causal effects of NMOSD on inflammatory proteins.

Supplementary Table S9: Sensitivity analysis results of causal effects of NMOSD on inflammatory proteins.

# Supplementary Figures

**
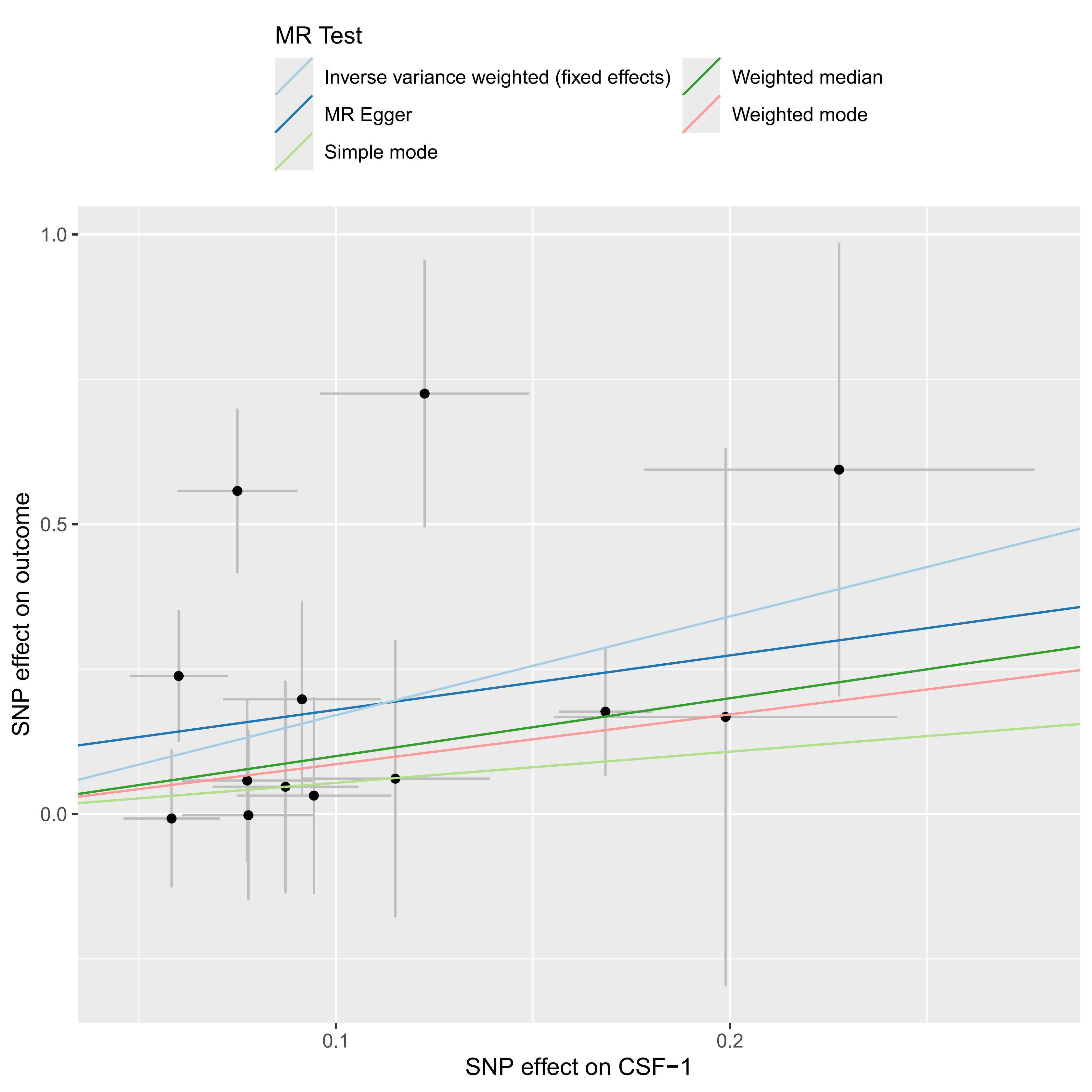
**

A


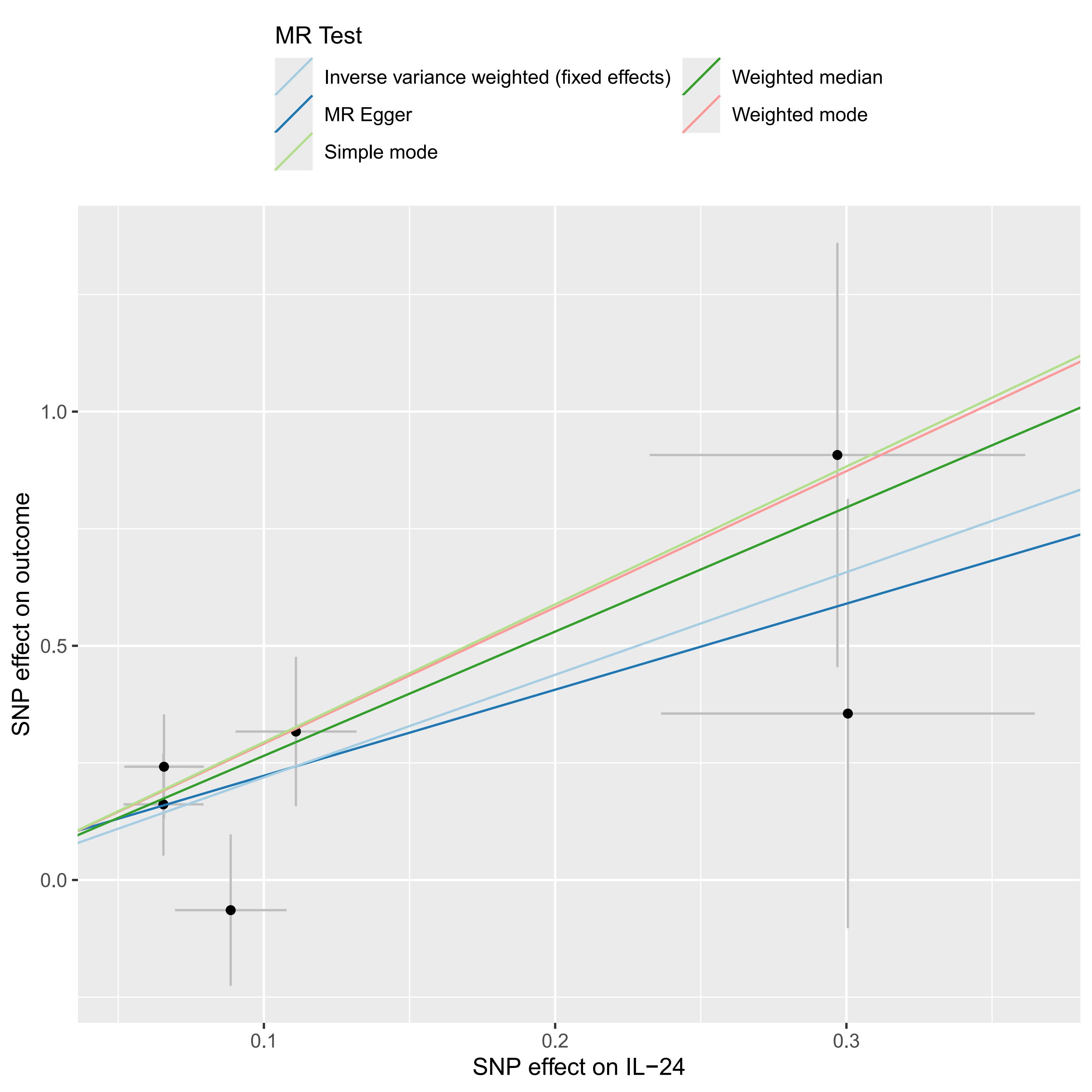


B

**Supplementary Figure 1.** Scatter plots for the relationship between the SNP effect size of causal inflammatory proteins and the corresponding effect size estimates of NMOSD. (A) Scatter plot for CSF-1 on NMOSD (B) Scatter plot for IL-24 on NMOSD


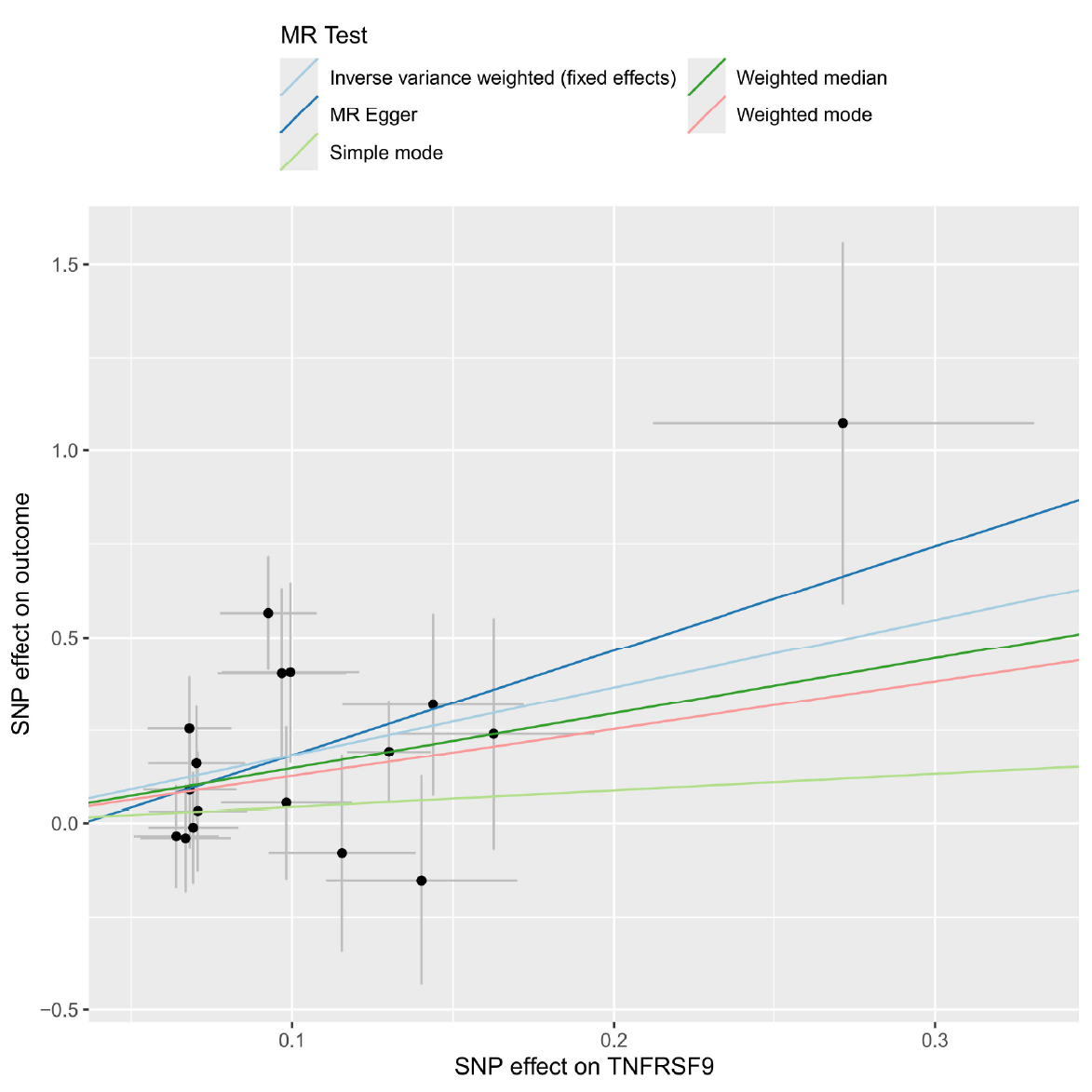


**Supplementary Figure 2.** Scatter plot for the relationship between the SNP effect size of causal TNFRSF9 and the corresponding effect size estimates of AQP4+NMOSD.


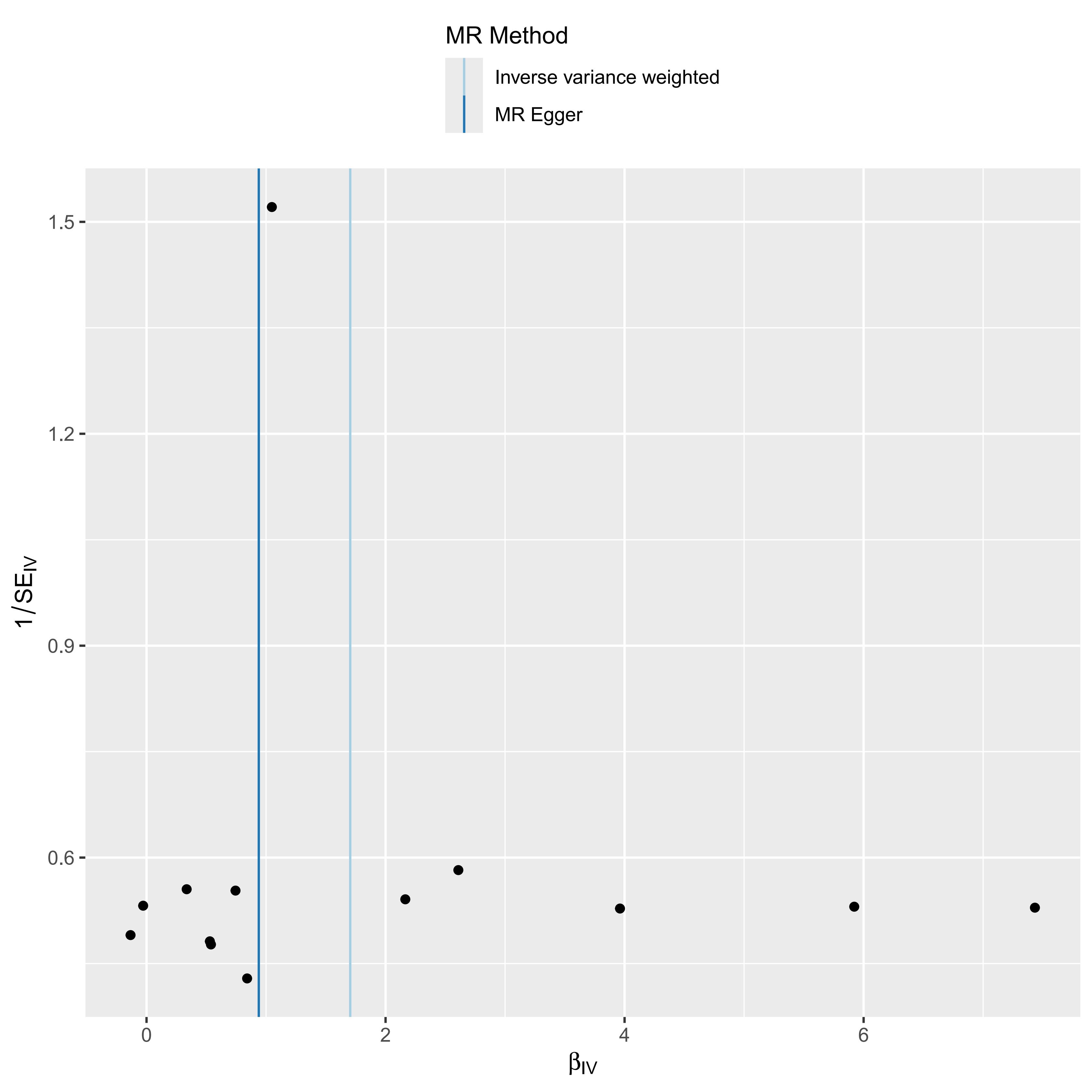


A


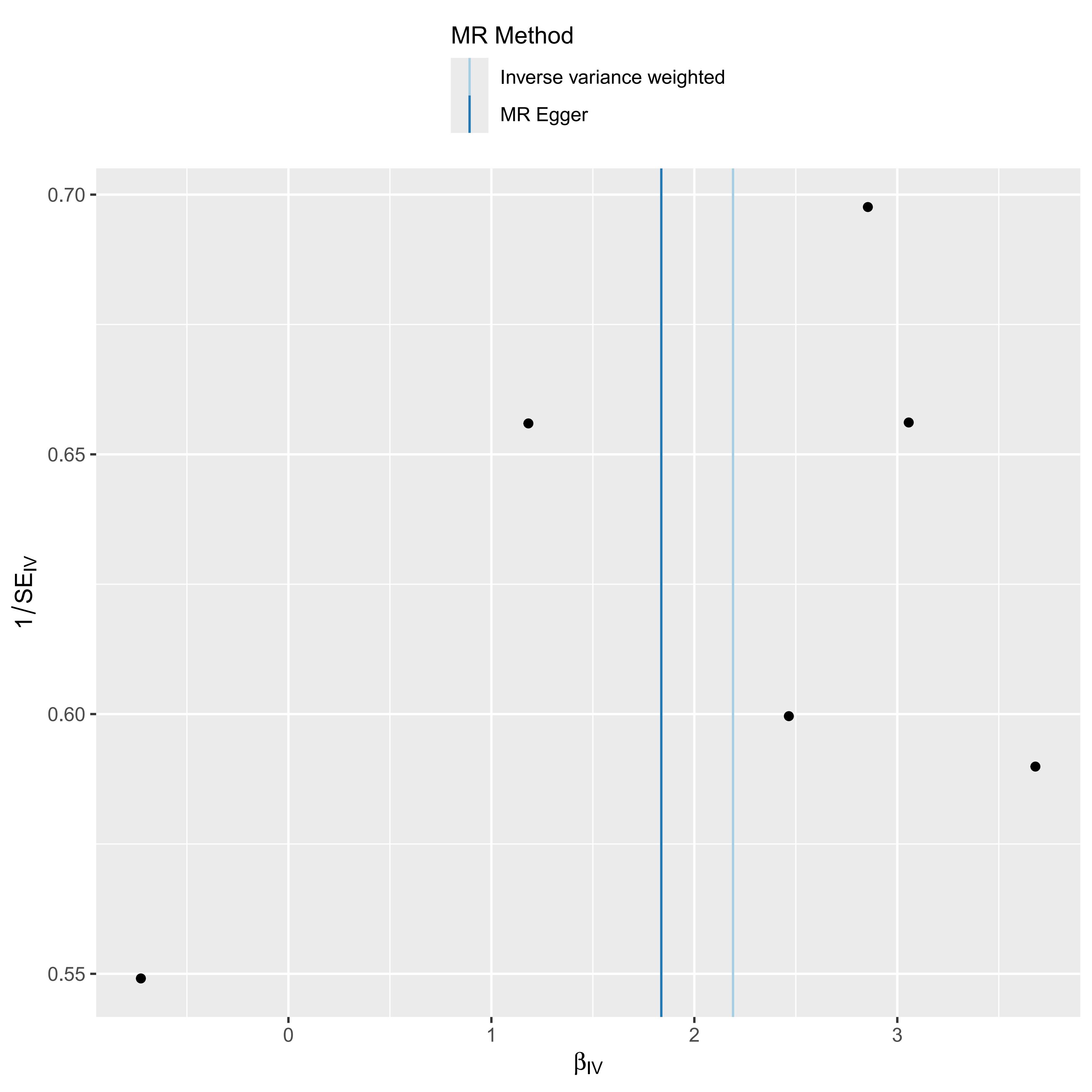


B

**Supplementary Figure 3.** Funnel plots between inflammatory proteins and NMOSD. (A) Funnel plot between CSF-1 and NMOSD (B) Funnel plot between IL-24 and NMOSD


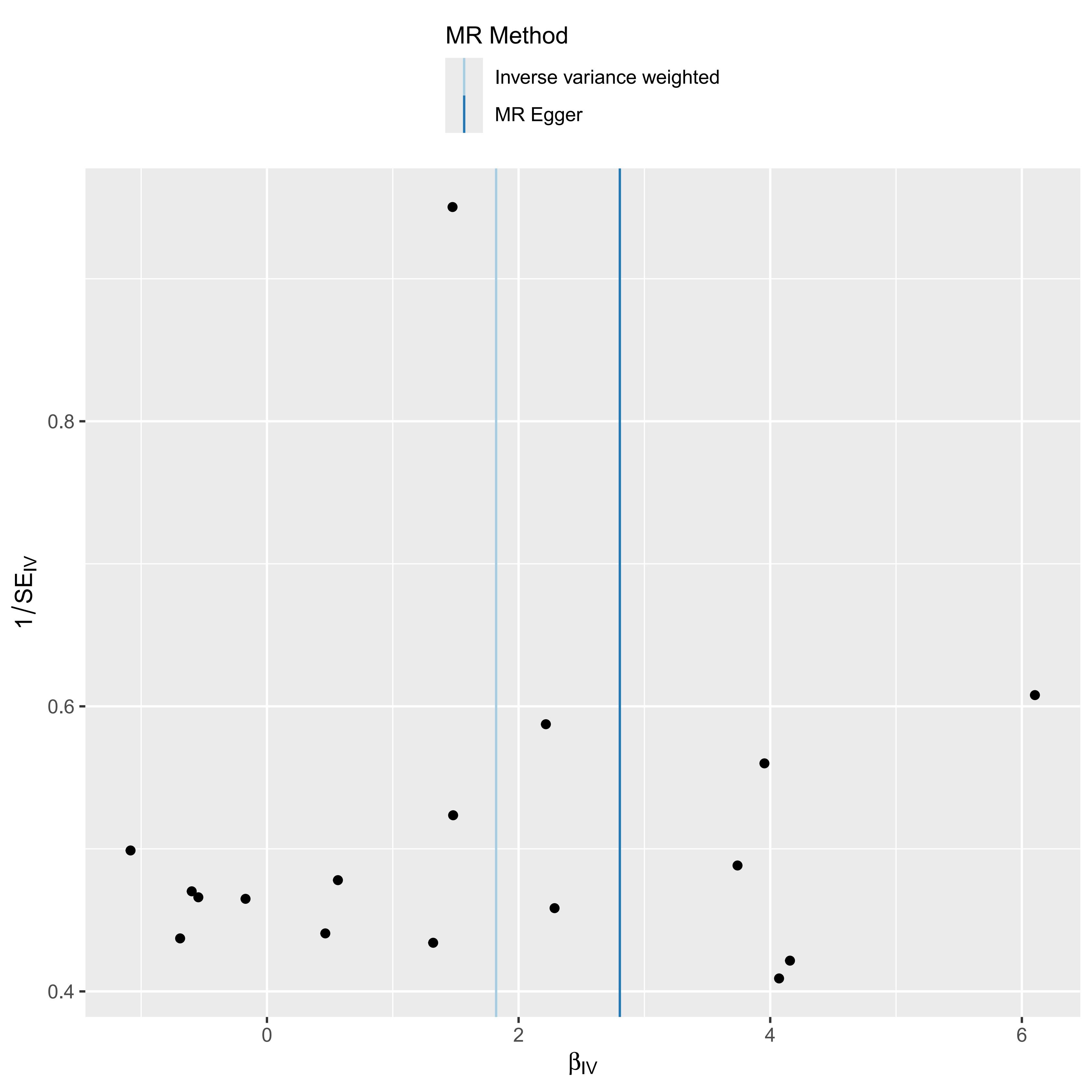


**Supplementary Figure 4.** Funnel plot between TNFRSF9 and AQP4+NMOSD.


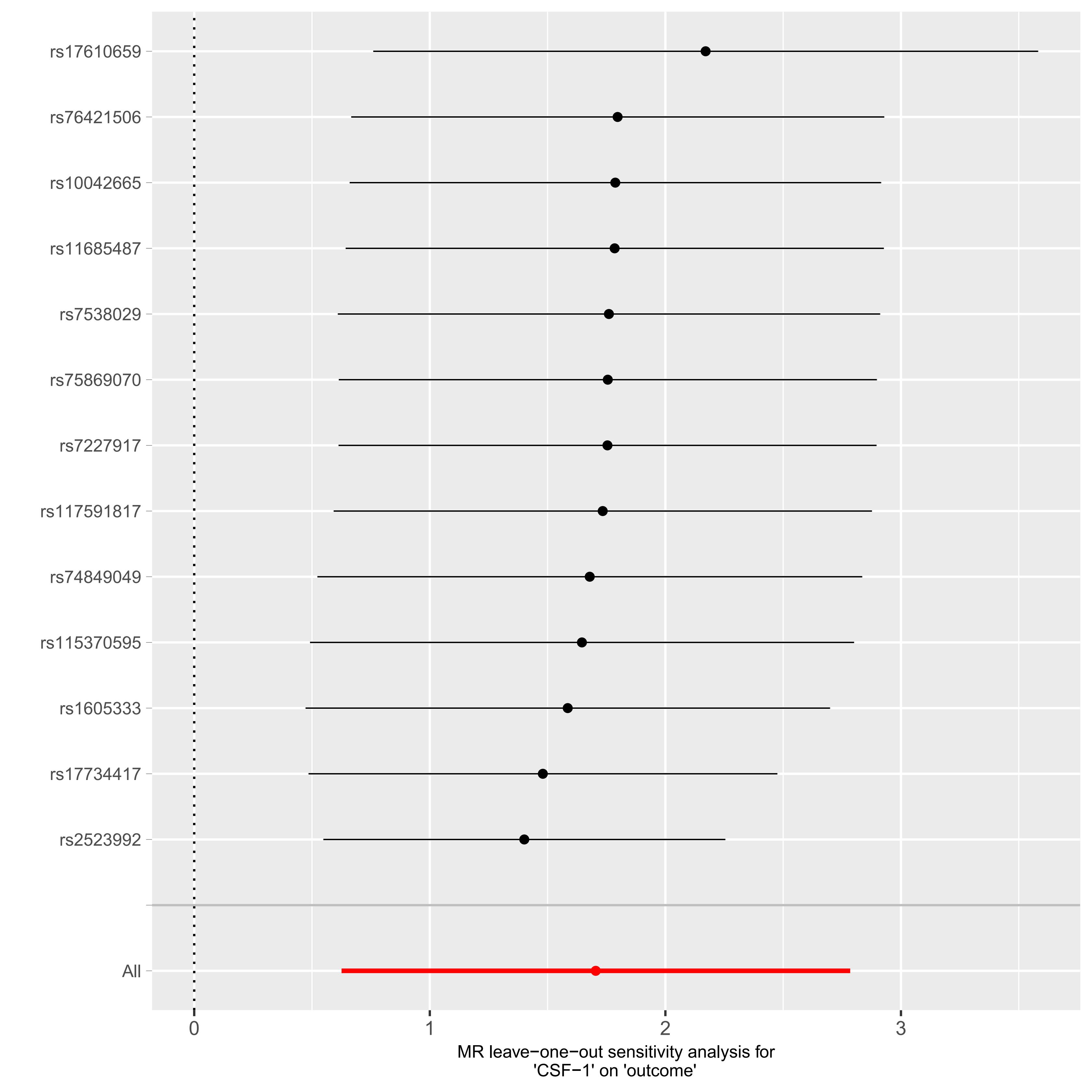


A


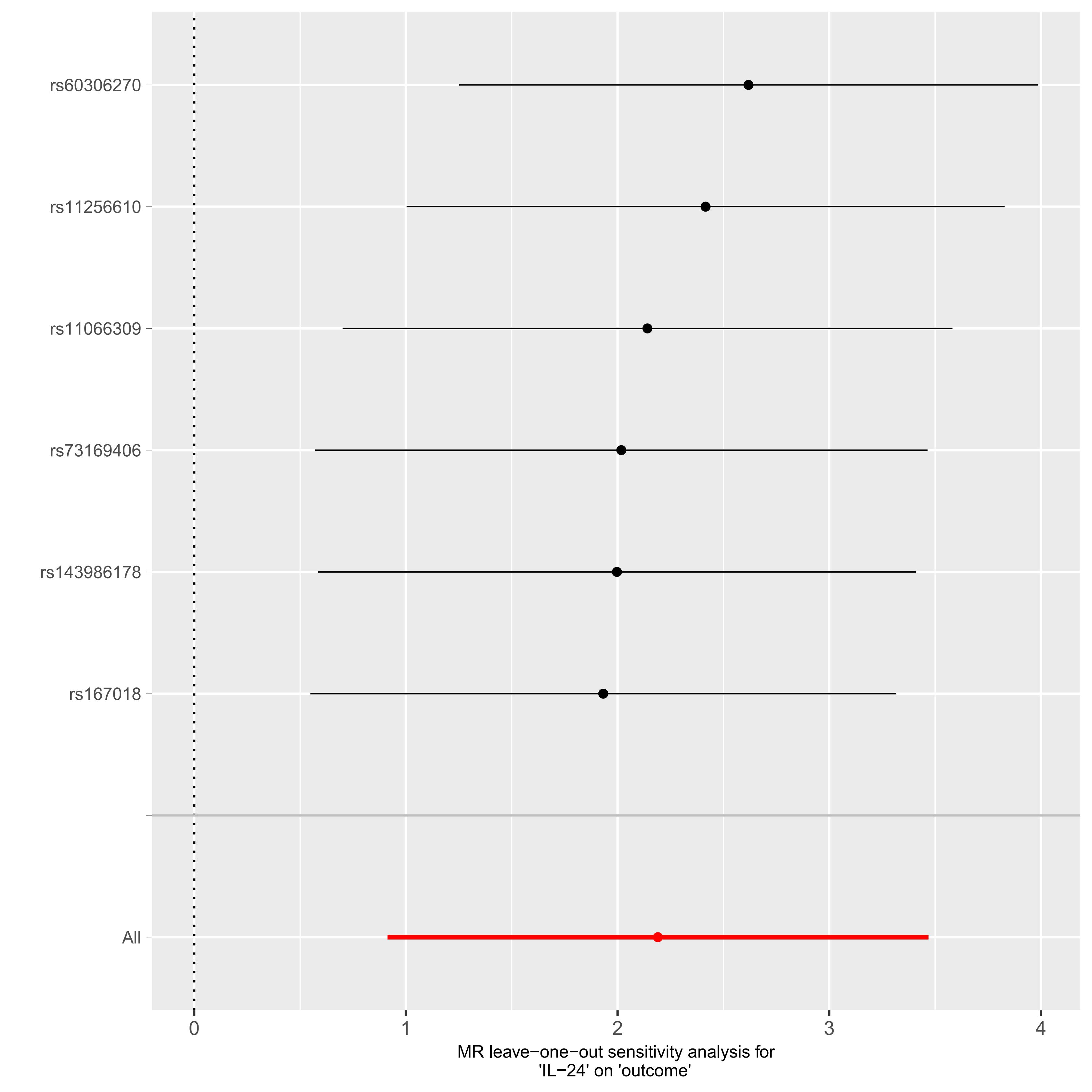


B

**Supplementary Figure 5.** Leave-one-out forest plots analysis of IVW estimates to assess the causal relationship between inflammatory proteins and NMOSD. (A) Leave-one-out forest plot CSF-1 and NMOSD (B) Leave-one-out forest plot between IL-24 and NMOSD

**
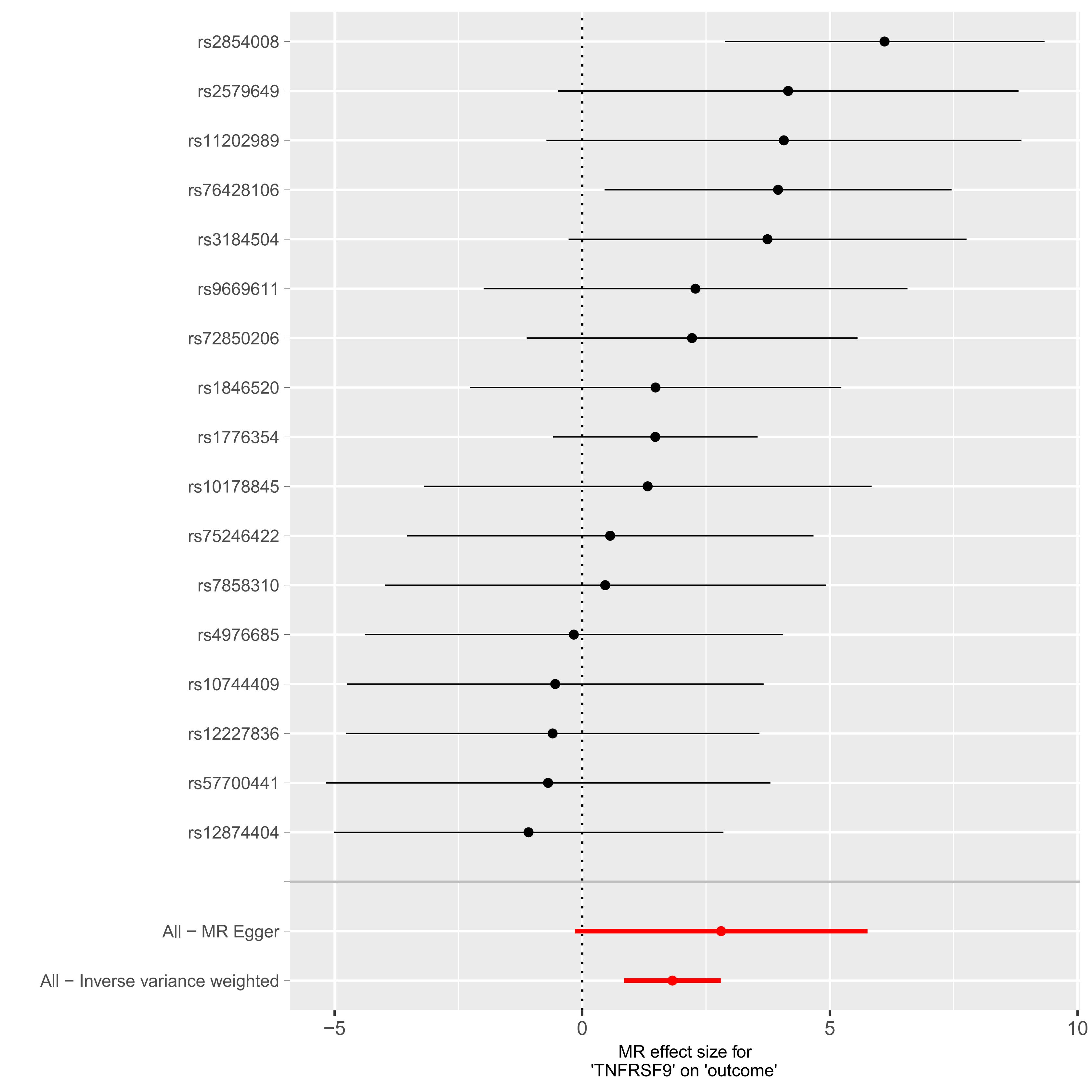
**

**Supplementary Figure 6.** Leave-one-out forest plot analysis of IVW estimates to assess the causal relationship between TNFRSF9 and AQP4+NMOSD.
